# The effect of flexible joint-like elements on the adhesive performance of nature-inspired bent mushroom-like fibers

**DOI:** 10.3762/bjnano.9.268

**Published:** 2018-11-19

**Authors:** Elliot Geikowsky, Serdar Gorumlu, Burak Aksak

**Affiliations:** 1 Mechanical Engineering, Texas Tech University, Lubbock, TX 79409-1021, USA; 2 Departmento Ingeniería Metalúrgica y de Materiales, Universidad Técnica Federico Santa María, Valparaíso, Chile

**Keywords:** bent fibers, bioinspired dry adhesives, gecko adhesion, joint-like element, mushroom-like fibers

## Abstract

Many organisms rely on densely packed, tilted and curved fibers of various dimensions to attach to surfaces. While the high elastic modulus of these fibers enables an extremely large number of fibers per unit area, where each fiber stands freely without sticking to its neighbors, the tilt/curvature provides them with the compliance and the directional adhesion properties to attach strongly and efficiently to a surface. Recent studies have revealed that many of such organisms also feature materials with a graded elastic modulus that is tailored towards improving the contact area without sacrificing the fiber density. In particular, for male ladybird beetles, research has shown that the adhesive setae feature a material gradient such that the elastic modulus of the material at the junction between the stalk and the divergent distal end is close to minimum. This soft material acts like a flexible joint, improving the bending compliance of the tip. Here, we mimic this feature using tilted, mushroom-like, stiff fibers comprised of a stiff stalk of elastic modulus 126 MPa, a softer tip of elastic modulus 8.89 MPa, and a joint-like element of elastic modulus 0.45 MPa (very soft), 8.89 MPa (soft), or 126 MPa (stiff) in between. The results from load–drag–pull (LDP) experiments performed along (gripping) and against (releasing) the tilt direction indicate that the soft and the very soft joint fibers performed superior to the stiff joint fibers and maintained directionally dependent performance. The soft joint fibers achieved up to 22 kPa in shear and 110 kPa in pull-off stress in the gripping direction, which are twice and ten times higher than that in the releasing direction, respectively. A model to optimize the elastic modulus of the joint-like elements to enable sliding without peeling of the tips has been proposed.

## Introduction

Most natural organisms that rely on temporary adhesion to surfaces for survival do so using tiny, densely packed fibers [1–2]. These fibers vary in dimension and material properties depending on the organism that bears them. They form an adhesive contact with the opposing surface, cumulatively providing a large enough force to support the organism’s body weight [3–4]. The strength of adhesion primarily depends on two quantities, the compliance of the fiber array as well as the number of fibers per unit area [5–7]. One important material property which influences these adhesive properties the most is the elastic modulus of the material forming the fibers [5]. For instance, in geckos, which utilize a hierarchical fiber structure ranging from millimeter-scale lamellae to micrometer-scale setae, to the nanometer-scale spatulae at the contact level, fibers are made from beta keratin [8] which has an elastic modulus of 1–4 GPa [9]. To enhance performance, given the high elastic modulus, all of the hierarchical levels forming the adhesive patch of the gecko are tilted rather than vertically aligned [10]. This tilt, in addition to enhanced performance [11], equips the gecko with directional adhesion properties as shown by Autumn et al. [12]. When they tested setae using a load–drag–pull (LDP) experiment, they found that setae exhibit very high interfacial shear and tension when dragged along the direction of the tilt. Opposite to the tilt direction, low shear and compression was measured. They called this phenomenon frictional adhesion suggesting that the adhesive engagement between the gecko’s foot and the surface is enabled only when it pulls the foot in the direction of the tilt, a consequence of the natural walking/running motion of the gecko.

Inspired by the directional adhesion features attributed to the tilted setal arrays of the gecko, considerable attention has been paid to fabricating various tilted fiber/pillar arrays and studying their adhesive performance both theoretically and experimentally [13–22]. Murphy et al. [16] obtained a similar anisotropy ratio in shear to the gecko setae and observed adhesive engagement only when the fibers were moved in the same direction as the tilt angle. They observed that the angle of the tip rather than the stalk played an important role in obtaining directional properties.

In the work by Wang [18], a method to produce slanted functionally gradient micropillars was proposed, whereby a magnetically assisted technique was applied to manage the compliance of the slanted fiber. By testing different scenarios, LDP results have shown the significant influence of the stiffness gradient in the robustness of the adhesion and the adaptability of the contact. Parness et al. [23] fabricated one of the first synthetic structures which performed similar to a gecko. While their adhesion was significantly lower than what has been observed with geckos, their structures exhibited very high friction in the direction along the tilt of fibers, termed here as gripping. Also, low adhesion allowed for an easily removable bio-inspired adhesive. Most of these works have featured softer fibers of monolithic construction, utilizing polymers with elastic moduli in the range 1–10 MPa. While it is desirable to use stiffer materials for fiber construction to improve fiber density and durability, only nanometer-scale fibers show adhesion when made from stiffer materials. Some examples can be found in the literature for carbon nanotubes [24–26] and stiff thermoplastic materials [27]. On the other hand, micrometer-scale fibers (i.e., fibers with diameter larger than 5 µm) do not adhere even to smooth surfaces as they lack the necessary contact compliance [28]. Composite fibers where the tip is softer than the stalk have shown tremendous promise in enhancing adhesion to both smooth and rough substrates [18,29–30]. Although the elastic modulus of the materials utilized for fiber fabrication do not reach that of the setae until a high aspect ratio, highly tilted fibers can be fabricated reliably to enhance compliance.

**Figure 1:**
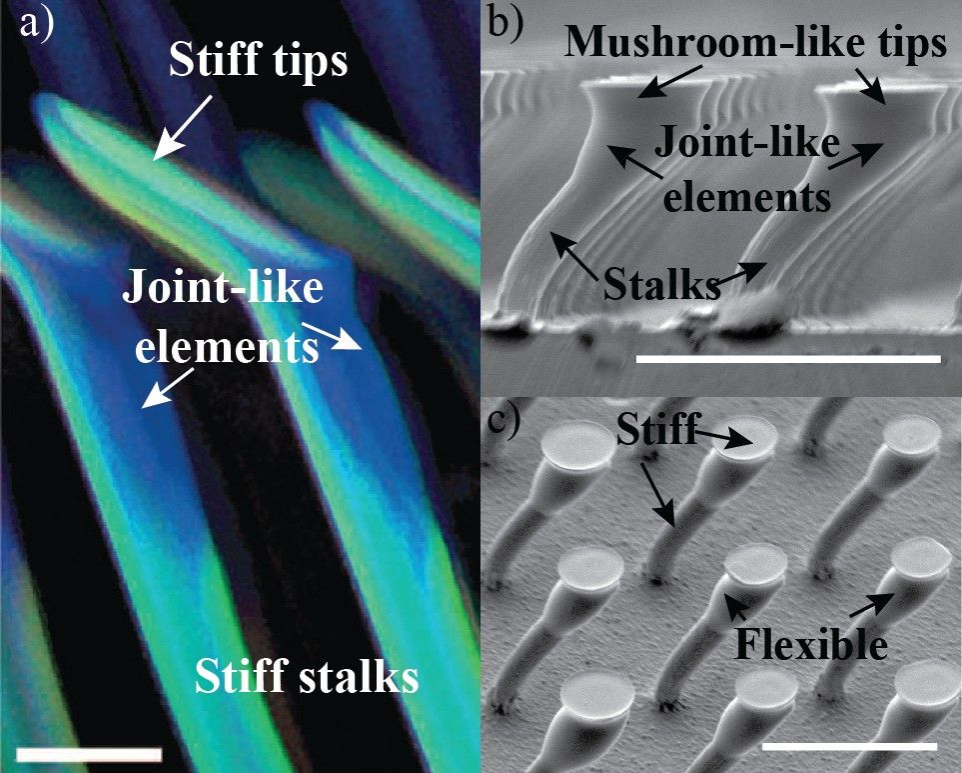
a) Confocal laser scanning microscopy (CLSM) of a lateral view of discoidal (mushroom-shaped) adhesive hairs in a male ladybird beetle. Differences in the autofluorescence indicate the presence and distribution of different materials. Blue regions (transitions from the hair shaft to the tip structure) indicate portions of the soft, rubber-like protein resilin. Light blue regions (hair shaft and discoidal tip structure) mainly consist of stiffer chitinous material. Adapted from [32]. b) Scanning electron microscope (SEM) images of synthetic, polymeric, bent fibers inspired by the adhesive hairs of the beetle, showing joints between the stalks and mushroom-like tips. c) Array of composite, synthetic fibers with stiff stalks and tips, and a joint in between fabricated using a softer material. Scale bars, 5 µm in a), 100 µm in b) and c).

In composite fibers, tip articulation could be critical for performance. In some biological attachment systems, such as the male ladybird beetle (*Coccinella septempunctata*), a joint of soft material between the setal stalk and the tip of an individual fiber has been discovered [31]. This feature, termed as a joint-like element, is evidenced by the difference in color of this joint from the stalk or the tip (see Figure 1a). The joint-like element is believed to be more flexible and equips the fibers with the necessary articulation to better adapt to the target surface [32]. This joint can consist of either a more flexible material than the stalk or a local geometrical constriction, which can provide robust adhesion independent of the direction of the applied load [32–33]. It can reduce the normal stress and alleviate peeling at the interface because the joint flexibility leads to a smaller resultant bending moment when compared to a monolithically constructed fiber.

Inspired by the construction and the material composition of beetle setal arrays as shown in Figure 1a, bent mushroom-like fiber arrays are fabricated from polyurethane materials using a stiff stalk, a softer tip, and joint-like elements with varying elastic modulus as shown in Figure 1b. Using these fibers, the effect of the elastic modulus of the joint between the stalk and the tip on adhesion and friction is investigated. All the fiber array stalks and the tips are made of polyurethane with elastic modulus *E*_s_ = 126 MPa and *E*_t_ = 8.89 MPa, respectively. The polyurethane used to fabricate the stiff stalks has a considerably higher elastic modulus that what has traditional been utilized for bioinspired microscale fibrillar adhesives to date. The fabrication technique to generate high aspect ratio, tilted fibers enhances compliance and facilitates the use of such a stiff polymer. The joint stiffness was controlled using three polyurethane materials of elastic moduli *E*_j_ = 0.45, 8.89 and 126 MPa as the joint material. The final array of the composite fibers consisted of a stiff stalk and tip linked by a soft joint as shown in Figure 1c. Friction and adhesion are measured as a function of initial compressive load (preload) using load–drag–pull (LDP) experiments. Fibers arrays were dragged in the direction of tilt (i.e., gripping direction) and against the tilt direction (i.e., releasing direction) to assess directional dependence of adhesion and friction.

## Results and Discussion

### Bent fibers with joint-like elements

Fibers of 20 µm diameter and 85 µm length were used to produce the bent fibers. They were evenly distributed in a 2.8 × 2.8 mm acrylic peg with a center-to-center distance of 60 µm, resulting in a peg with approximately 2,100 fibers. Mushroom-like caps of around 50 µm diameter were produced for both stiff and soft joint fibers.

**Figure 2:**
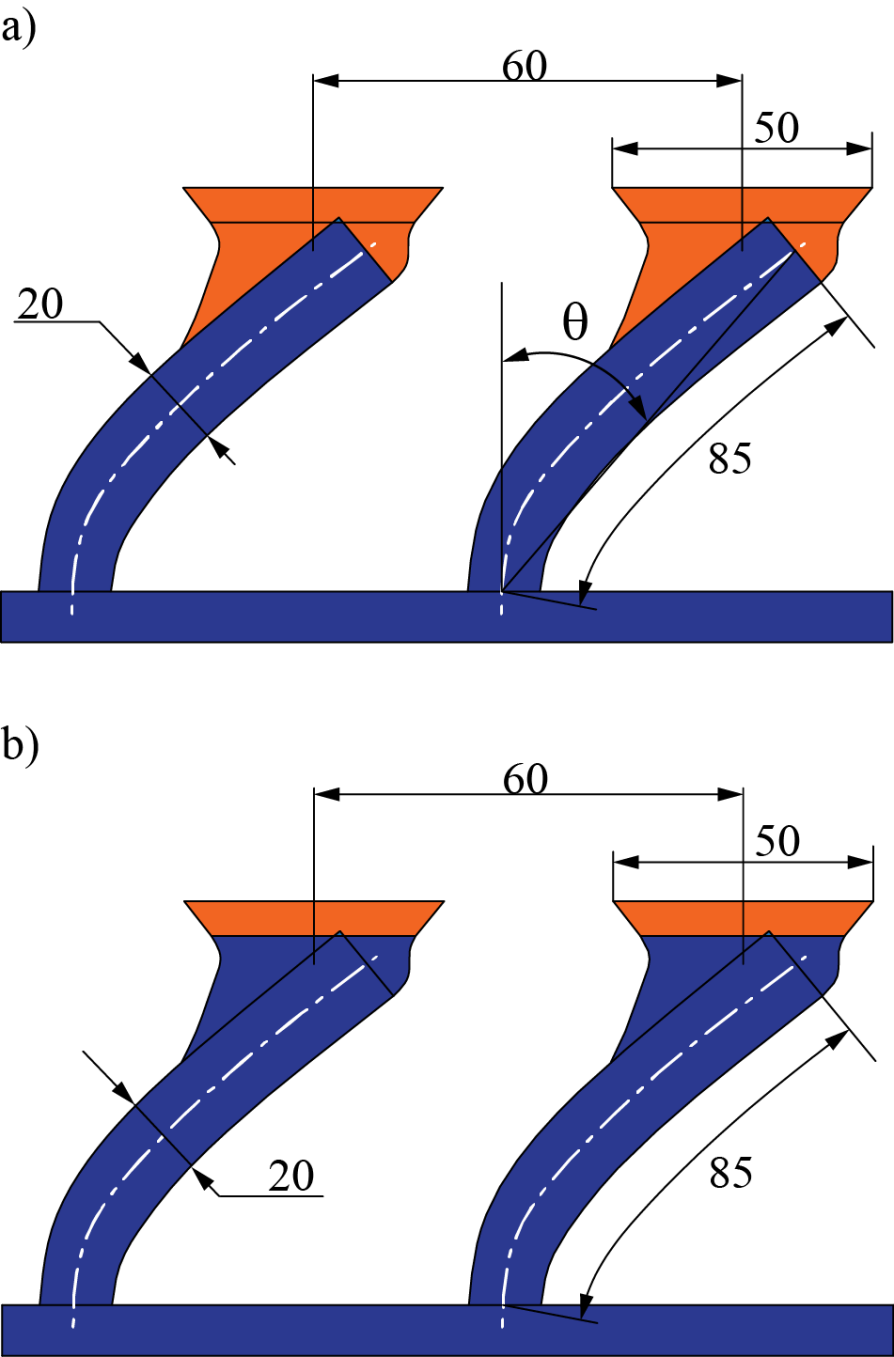
Geometrical characterization of the fibers used in this research detailing the zones where soft (orange) and stiff materials (blue) are used. Dimensions are given in µm. a) Soft joint fibers use soft material in both caps and joint-like elements while a stiffer material is used for the stalks. b) Stiff joint fibers use soft material only for the mushroom-like caps while stalks and joint-like elements are made of stiff material.

Although any desired angle of curvature can be produced by the proposed method, in this research, fibers of an effective angle of 57° were used. The effective tilt angle is defined in the process of bending the fibers, before the caps are constructed. For this reason, the effective tilt angle is measured between the base and the tip of the fiber stalk (Figure 2a).

### Load–drag–pull curves in gripping direction

**Figure 3:**
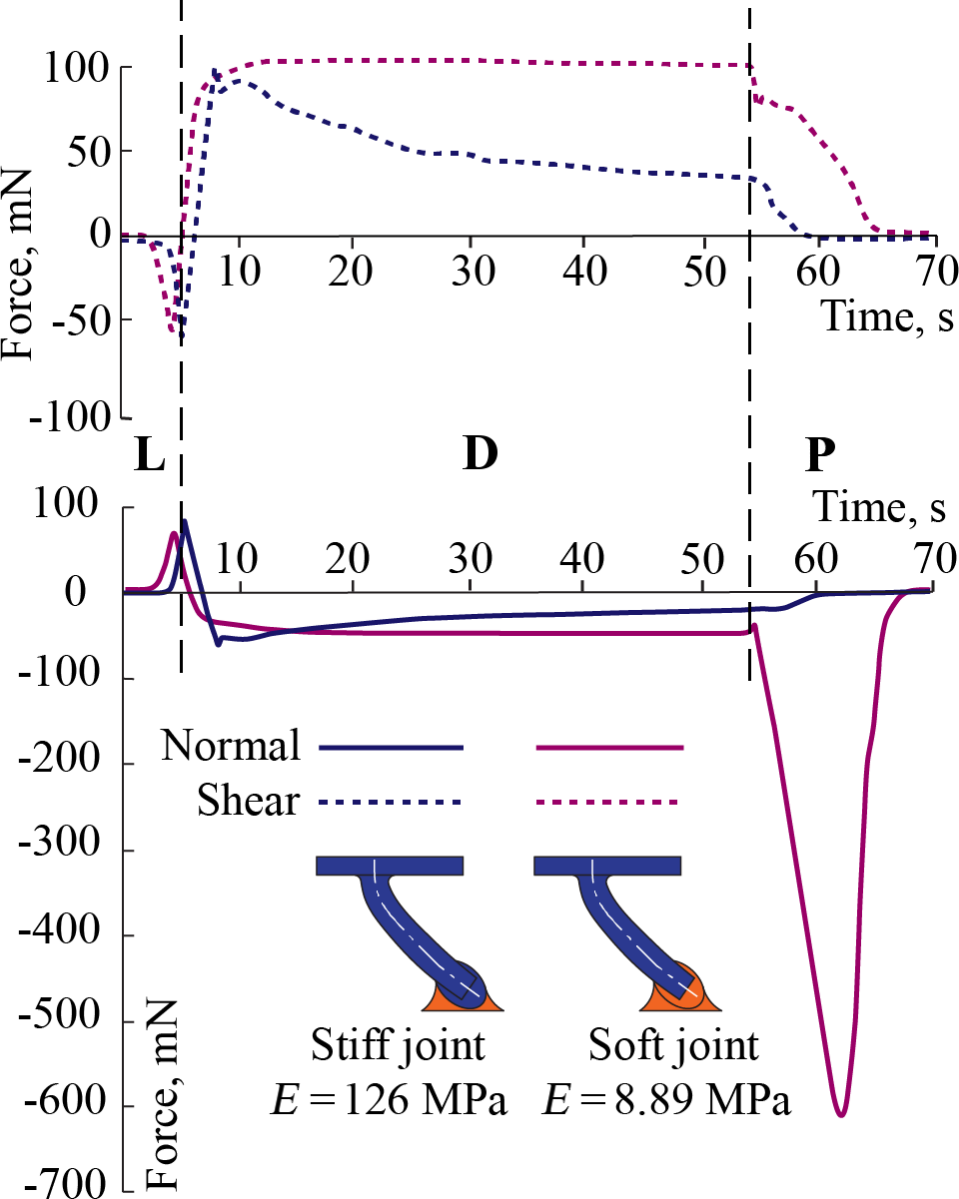
Shear and normal forces for soft (magenta) and stiff (blue) joint fibers in a load–drag–pull (LDP) test in gripping direction. L (loading), D (dragging) and P (pulling) regions are denoted in the graph. Both types of fibers react similarly until the loading step. At sliding, a continuous decrease in force is observed when stiff joints are used for the fibers. For fibers with softer joints, both forces stay constant while sliding and a significant pull-off force is obtained during the pull stage.

Sample data from two cases are given in Figure 3 to show the effect of joint flexibility on the normal and shear loads in an LDP test. The solid lines represent the normal and the dashed lines represent the shear forces. The force results are not plotted against displacement because the test has two directions of movement, parallel in shear and perpendicular when approaching and when receding. These displacements cannot be appropriately represented in one two-dimensional graph. Instead, a clearer representation of the physical phenomena can be obtained when plotting against time.

**Figure 4:**
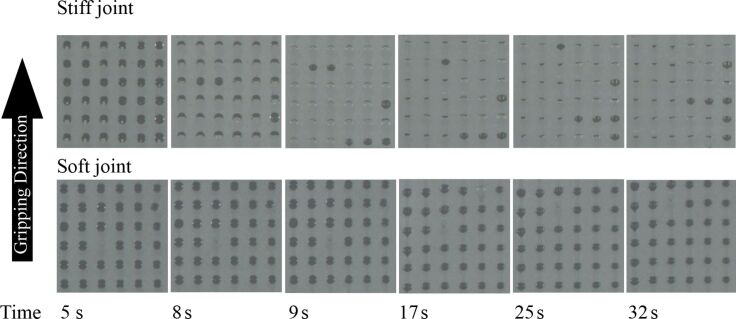
Sequence of images showing the contact of the fibers with the substrate while sliding in the gripping direction for stiff and soft joint fibers. Preload is reached at 5 seconds and sliding in the gripping direction starts. Three seconds later, stiff joint fiber caps begin to detach, while for the same period of time, soft joint fiber caps remain attached. After 9 seconds, stiff joint fiber caps continue to detach while soft joint fiber caps remain in contact.

In the “loading” stage, the sample approaches the substrate and contact is initiated. The relative normal displacement stops after a desired preload is reached, indicated by a positive peak in the normal force curve, observed at about 5 seconds after the start of the test as shown in Figure 3. This is immediately followed by the “dragging” stage, where two phases can be seen. An initial phase, where the fiber stretches due to the lateral force, shows close to straight lines in both shear and normal force. The normal force gradually switches from positive (compression) to negative (adhesion). A second phase depicts the relative displacement of the fibers with respect to the target surface once the fibers have been stretched. In this phase of dragging, stiff and soft joint fibers behave in a completely different way. While both samples exhibit adhesion during sliding, soft joint fibers remain attached to the target surface while sliding, resulting in a constant shear and normal force in Figure 3. On the other hand, the decreasing response in both shear and normal force for stiff joint fibers suggests that the fibers gradually lose contact with the substrate during dragging. After about 55 seconds, once a desired relative transverse displacement is achieved, the samples are retracted in normal direction from the substrate in the “pull” stage of the LDP experiment, exhibiting a pull-off peak. This negative peak is recorded as the pull-off force. The relatively large pull-off force observed in soft joint fibers suggests minimal loss of contact. 27 seconds of the contact performance of the tips while sliding in the gripping direction is shown Figure 4 for stiff and soft joint fibers. After the maximum preload is reached at about 5 seconds, the stiff joint fibers gradually detach when they slide in the gripping direction. The rigid nature of these fibers enforces the misalignment of the tips when sliding, producing the consequent detachment, which does not necessarily result in damage of the fibers. In contrast, the higher bending compliance of soft joint fibers keep them attached to the substrate while sliding, allowing superior performance in terms of pull-off force.

**Figure 5:**
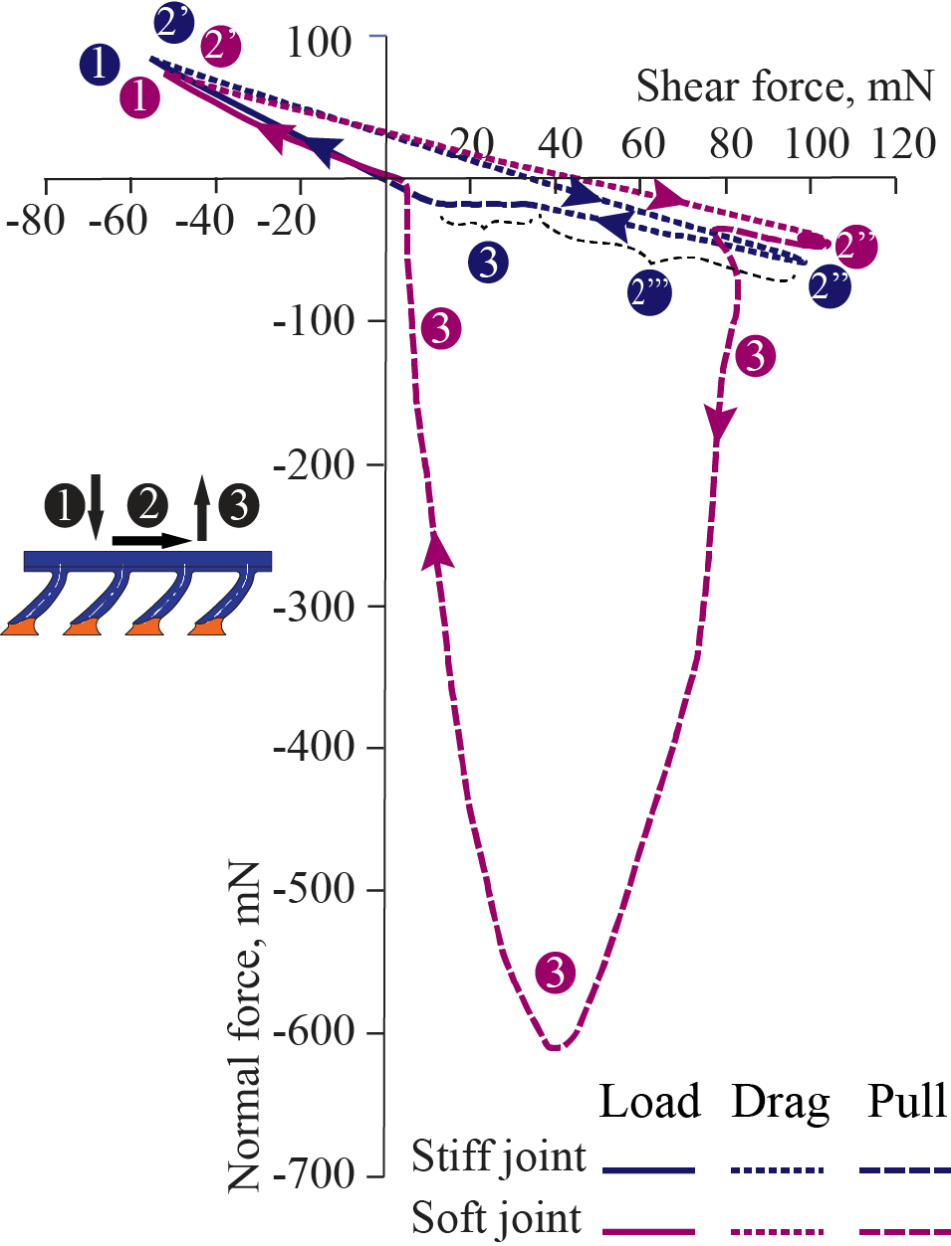
Normal versus shear force for soft (magenta curve) and stiff (blue curve) joint fibers in a LDP test in the gripping direction. Both samples react similarly at the loading stage – point up to 1 – and prior to sliding starts at point 2’’. The forces remain steady when soft joint fibers are dragged, but both shear and adhesive forces decrease for the stiff joint fibers. The pull-off section – points 3 – shows no adhesion for stiff joint fibers and a large pull-off peak for soft joint fibers.

Similar observations can be made in shear versus normal force plots. Figure 5 shows one complete cycle of the LDP test in the gripping direction for stiff and soft joint fibers. The test starts at the origin of the graph, and the predetermined preload is reached at point 1 of the graph. The dragging section of the test begins at point 2’ immediately after preload is reached. The initial phase of stretching results in close to straight lines in the plot, which implies an increase in the shear force but also a partial release of the normal force initially set at the loading stage. The close to straight curves at the loading stage and for the initial phase of dragging exhibit smaller slopes for soft joint fibers than for stiff joint fibers. The increase in bending compliance of the soft jointed fibers could explain this trend. The second phase of the dragging stage begins when fibers slide with respect to the target surface, which does not occur until the maximum value of shear is reached at point 2’’. The different response of the two samples is easily observable in the plot. The saturated zone of points observed at point 2’’ of soft joint fibers reflects constant normal and shear forces; the curve for stiff joint fibers, labeled as 2’’’, returns towards the origin of the graph due to the decrease in both normal and shear forces, indicating a gradual loss of contact with dragging distance. The dissimilar performance in sliding for both samples implies a completely different response for the pull-off force. The pull-off section of the test is denoted as 3 in the plot. While soft joint fibers exhibit a large pull-off force, stiff joint fibers exhibit null pull-off force, which is observed in the small portion of curve indicated by the blue point 3 in Figure 5.

### Performance in gripping and releasing directions

**Figure 6:**
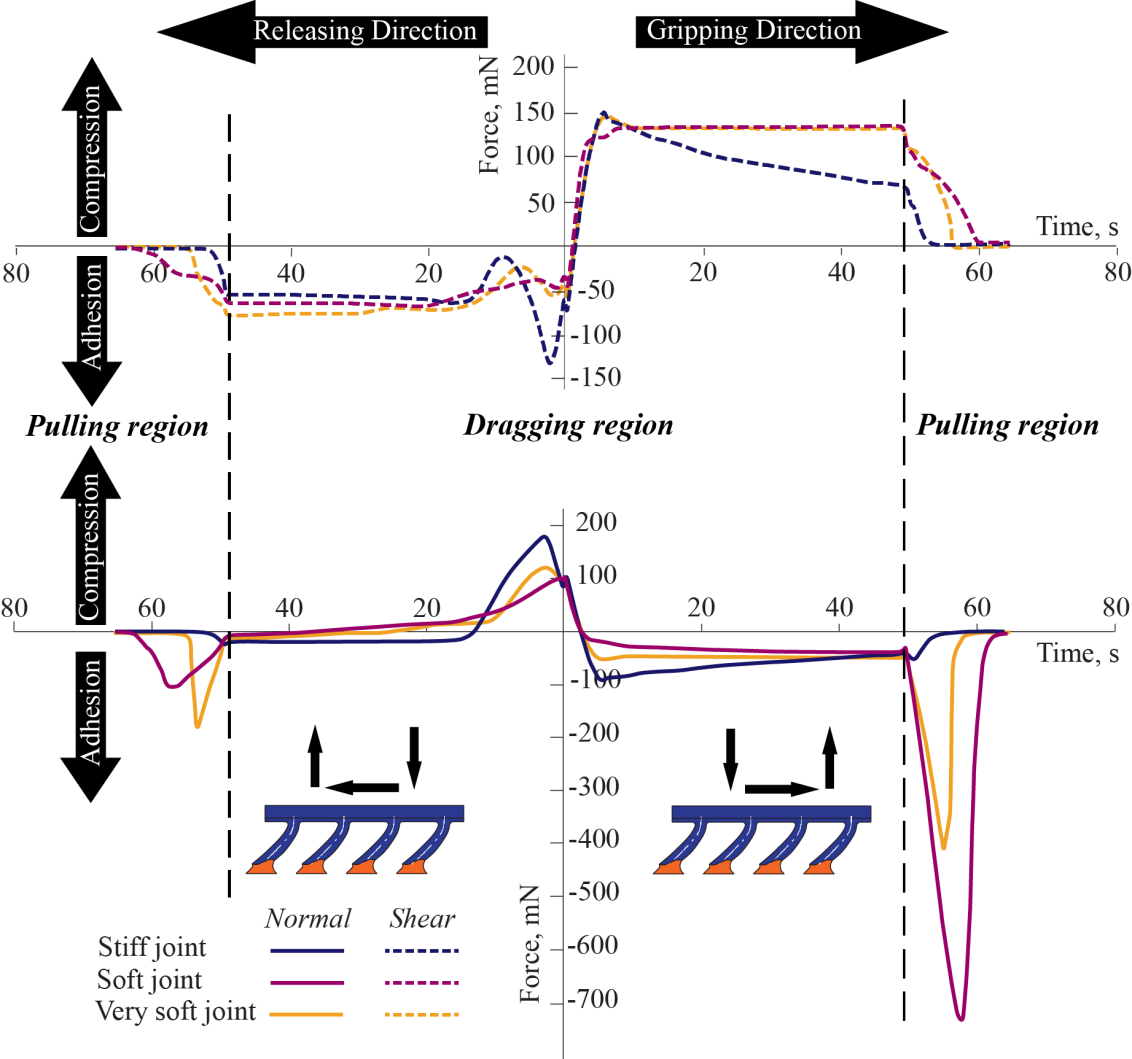
Shear and normal forces in LDP tests along releasing and gripping directions for stiff, soft, and very soft joint fibers. The dragging stage lasts 50 seconds to cover 500 µm lateral displacement in each direction. Time axis labels are included at the bottom of the plot. An initial vertical preload of 100 mN is applied for all three samples. Positive normal force represents compression, negative normal force represents adhesion of the fibers. Shear forces opposite to the sample displacement direction are positive when dragging in the gripping direction and negative in the releasing direction. Stiff joint fibers have decreasing shear and adhesive forces in the gripping direction, whereas similarly even forces are obtained from soft and very soft joint fibers in the gripping direction. Shear force in the gripping direction is roughly twice the shear in the releasing direction for soft and very soft joint fibers, indicating a shear force anisotropic ratio of 2:1.

An important aspect of natural curved/tilted fibers is the directional dependence of adhesion and shear. Similar anisotropy can be observed for the samples tested in this work as well (see Figure 6). All the samples exhibit higher shear forces in the gripping than in the releasing direction, the discrepancy being larger for the soft joint and the very soft joint fibers. All the samples show adhesion in the gripping direction. Their adhesion values are either lower or they are in compression in the releasing direction. There exists a pull-off peak for all the samples both in the releasing and the gripping direction. The pull-off forces are much larger for the soft and very soft joint fibers than the stiff joint fibers. In the dragging phase, where the fibers start sliding with respect to the substrate, the fibers slide steadily without showing a stick–slip behavior as evidenced by the smooth shear force curves. This behavior is in line with the performance of setae in an LDP test [12].

The role of the joint-like elements in adhesion and shear can be better understood if a wide range of preloads is used for the tests. A range of preloads between 10 mN to 200 mN is used to test each of the three samples. An ascending order in the application of preload is followed for both gripping and releasing directions. The tests are performed first in the gripping direction for all preloads followed by tests in the releasing direction. Each sample is tested once at a given preload.

**Figure 7:**
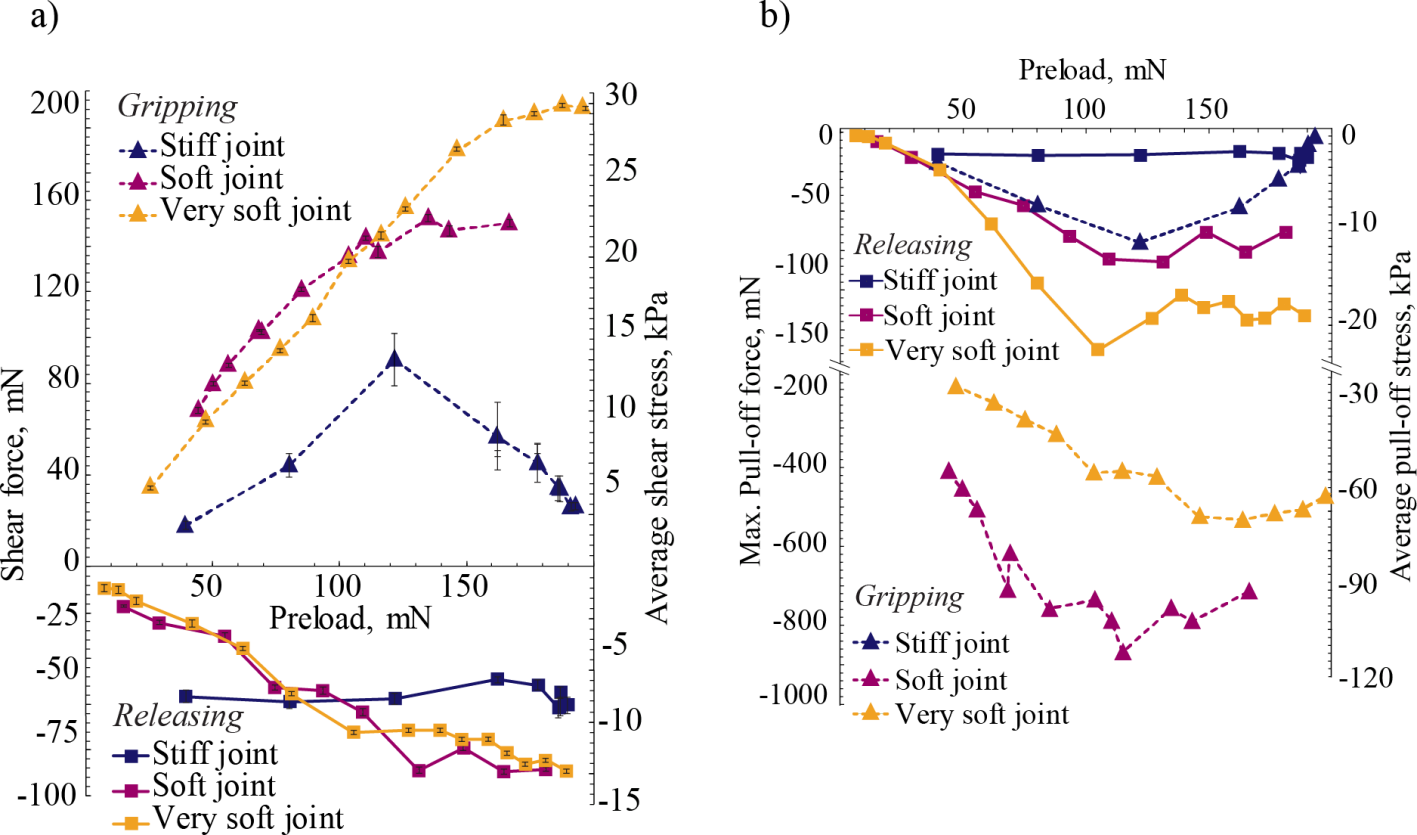
a) Shear force and average shear stress during the sliding phase of the dragging stage as a function of preload. Sliding is identified in the dragging region in Figure 6 as the portion of curves between 10 to 50 seconds. The results are in terms of average force and standard deviation within the described phase of the stage. Results in the gripping direction indicate reduced shear force in stiff joint fibers after a preload of about 120 mN, suggesting contact loss at higher preloads. Soft and very soft joints provide superior shear force and gradual increase until a saturation limit is reached, attributed to better tip contact even for higher values of preload. Enhanced bending compliance also helps in the releasing direction, an undesired consequence of softer joints. Both soft and very soft joint fibers exhibit higher shear in the gripping than the releasing direction. b) Pull-off force and stress as a function of preload. Soft and very soft joint fibers exhibit much higher pull-off stress than the stiff joint fibers, reaching as high as 110 kPa for the soft joint fibers. All samples exhibit higher pull-off force in the gripping direction. A pull-off force anisotropic ratio of up to 10:1 is observed between the gripping and releasing directions for the soft joint fibers.

Figure 7a shows shear force and average shear stress (i.e., the shear force divided by the apparent sample area) as a function of applied preload. The shear force values reported here are the average of the shear force values during sliding and the corresponding error bars of standard deviation. Positive values of shear force indicate shear in the gripping direction, negative values indicate shear force in the releasing direction. A proportional relationship, which leads to saturation at higher preloads, is observed between shear forces and preloads for soft and very soft joint fibers in both directions. Shear force saturates starting at a preload of 150 mN for soft joints. The saturation shear stress is higher for very soft joints, which is also reached at a higher preload of 200 mN. The ascending trends in the plots are an indication of the increase in the number of fibers in contact when the preload in increased. The magnitude of the response in shear of soft joint fibers suggests that shear force in the gripping direction is approximately twice as high in the releasing direction, providing an anisotropy ratio of 2:1 at the highest applied preload. This ratio is marginally higher for very soft joint fibers. The highest average shear stresses of 22 kPa and 30 kPa are obtained with the soft and the very soft joint fibers, respectively, at the highest applied preload for each sample.

Figure 7b shows the results for the largest adhesive force recorded when the sample is retracted from the substrate as can be observed in the pulling stage in Figure 6 for normal force, defined here as the pull-off force, as a function of preload. The same figure also includes pull-off stress calculated by dividing the pull-off force by the apparent area of the tested sample. Pull-off force mainly increases with preload for both the soft and the very soft joint fibers in both directions. The soft joint fibers exhibit the largest pull-off stress for all preloads, reaching as high as 110 kPa in the 100 mN to 120 mN preload range. Note that the pull-off force at this preload is approximately eight times the applied preload. A pull-off force ratio of up to 10:1 is observed between gripping and releasing directions for soft joints at the lowest preloads. This ratio is smaller at 8:1 for the very soft joint fibers at similar preloads.

Fibers with stiff joints exhibit inferior performance in adhesion compared to soft and very soft joint fibers, with maximum pull-off stress being limited to 10 kPa. Additionally, the difference in pull-off stress between the gripping and releasing directions is less pronounced when compared with the soft and very soft joint fibers. Similar to the results obtained with the gecko setae, all of the samples exhibit adhesion during sliding, which is a necessary condition for an adhesive pad bearing animal/robot to climb vertically.

### The relationship between normal and shear force

In their tests with an isolated seta, Autumn et al. [12] concluded that setae exhibit compression in the releasing direction where the shear force is proportionally related to this compressive force through the relation 
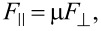
 where 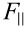 and 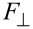 are the parallel and normal forces, respectively. For the purpose of this study, 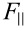 and 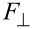 are measured at the moment of sliding, meaning within 10 to 50 seconds of the LDP test as can be seen in Figure 6 for the releasing and gripping directions. For each test a mean and standard deviation of parallel and normal forces are calculated. This result is in line with Coulomb friction [34]. In the gripping direction, where adhesion force is observed, they found that there is a relationship between adhesion (normal force) and friction (parallel force), independent of the magnitude of each force, such that


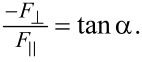


Here the angle α represents the critical angle of the setae at which sliding starts. They termed this phenomenon frictional adhesion.

**Figure 8:**
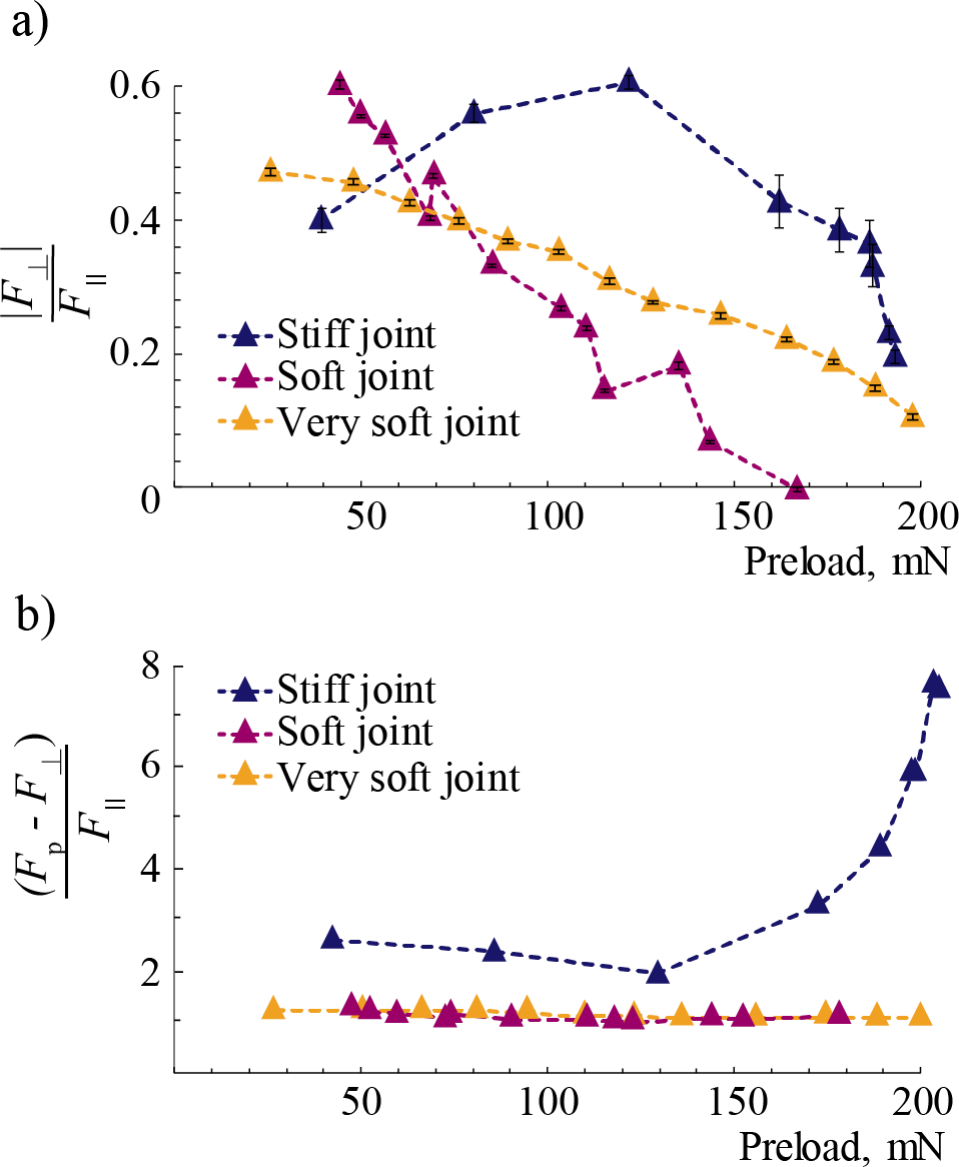
a) The ratio of adhesive to shear force during sliding in the gripping direction as a function of preload. The gradual decrease in this ratio for the soft and very soft joint fibers suggests a lower effective detachment angle with preload according to the frictional adhesion model proposed by Autumn et al. [12]. For stiff joint fibers, the ratio increases until fibers switch from full to partial contact. b) The ratio of the difference between preload and adhesion to the shear force in the gripping direction as a function of preload. An approximately constant relationship exists for the soft and very soft joint fibers for all the preloads, and for the stiff joint fibers until there is loss in both adhesion and shear.

In our tests, we observed no substantial adhesion or mostly compressive forces in the releasing direction. Frictional adhesion was primarily observed in the gripping direction. In Figure 8a, we plotted the ratio of adhesive force to shear force as a function of preload in the gripping direction. Unlike in the case of gecko setae, the results indicate that this ratio is not constant with preload for all three tested samples. This discrepancy, which is a consequence of the testing method, is expected as Autumn et al. used a constant preload in their experiments. The initial deflection of the fiber as well as the normal and shear stresses at the interface prior to the dragging stage all increase with preload. This higher deflection, provided the fiber tip starts sliding without peeling, will lead to a higher deflection angle (i.e., lower tan α) as shown in Figure 8a for soft and very soft joint fibers. We hypothesize that there is a constant relationship between the difference between preload *F*_p_ and adhesion force 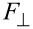, and shear force 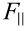 such that


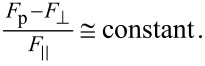


As shown in Figure 8b, when this ratio is plotted against preload, an approximately constant relationship is revealed for the soft and very soft joint fibers for all the preloads, and for the stiff joint fibers until there is loss in both adhesion and friction.

### Optimal joint flexibility

**Figure 9:**
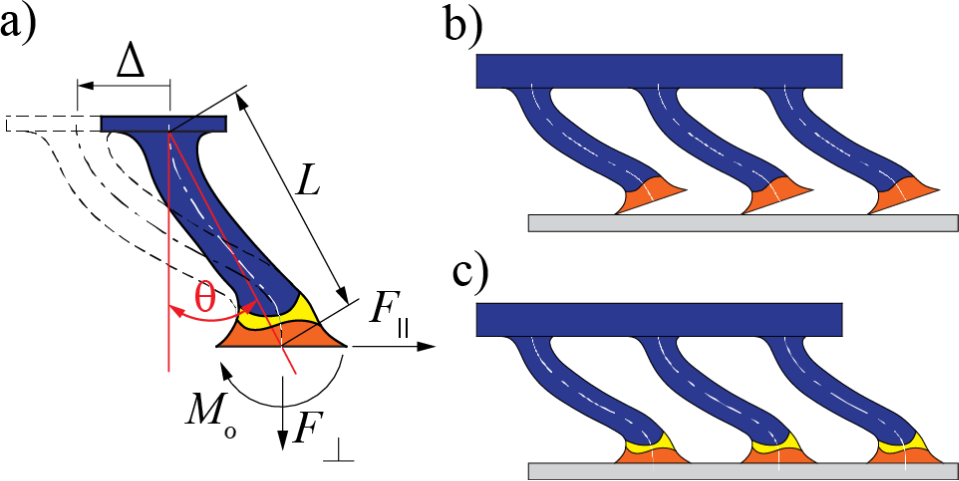
a) The forces at the fiber when dragged along the direction of tilt. b) Fibers start to peel from the substrate during sliding due to lack of tip articulation. c) Fibers start sliding without peeling from the substrate, aided by the added articulation with a flexible tip joint.

Figure 9a shows the forces and moment developed at the fiber when the fiber is dragged along its tilt direction. There are mainly two ways an adhesive fiber can fail. The most common failure mechanism is mode I, where a portion of the fiber tip, usually the trailing edge, peels away from the surface when it is dragged against it primarily due to the bending moment *M*_o_ at the tip, Figure 9b. The bending moment creates a tensile stress at the vicinity of the trailing edge. A large enough tensile stress causes the trailing edge to peel, leading to partial contact with the surface. A more desirable failure mechanism in synthetic adhesives is mode II, where the tip starts sliding with respect to the surface, maintaining full contact, Figure 9c. This mode of failure is achievable if the shear stress at the interface reaches an intrinsic interfacial shear strength before peeling takes place (i.e., normal stress reaches an intrinsic interfacial tensile stress).

The existence of a flexible joint between the tip and the stalk of a fiber aids in minimizing the bending moment at the tip. We can solve for *M*_o_ using compatibility as the problem is statically indeterminate. Let us model the flexible joint as a torsional spring of stiffness


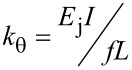


added to the end of the fiber, where *E*_j_ is the elastic modulus of the joint material and *f* is the thickness of the joint in terms of overall effective length *L* of the fiber, and *I* is the area moment of inertia of the fiber assuming constant cross-section for the entire composite fiber. Prior to peel, the boundary condition for the fiber is fixed–fixed. Assuming that the problem is linear, and for simplicity that the fiber is tilted but straight, the compatibility equation such that the null slope at the tip due to the fixed boundary condition is unchanged becomes

[1]
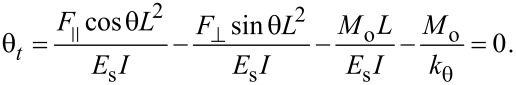


Here, *L* is the effective length of the fiber, θ is the effective tilt angle, *E*_s_ is the stalk elastic modulus. The first three terms in this equation represent the classical solution for the tip deflection angle and the last term accounts for the rotation of the flexible joint. In this equation we assumed that the deflection of the tip is predominantly caused by the bending moment *M*_0_. Assuming 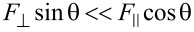 for a low bent angle θ, we omit the second term in Equation 1. Rearranging, one obtains *M*_0_ as

[2]
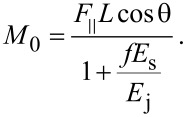


Equation 2 indicates that a flexible joint helps to reduce the bending moment at the tip. Without a flexible joint, the second term in the denominator would be zero (i.e., *E*_j_ = ∞ or *f* = 0) maximizing the bending moment at the tip. As the torsional stiffness of the flexible joint decreases, so does the bending moment at the tip. Let us define the bending moment in terms of a maximum tensile stress at the interface (σ_s_) using the flexure formula such that *M*_0_ ≡ φπ*c*^3^σ_s_. Here, *c* is the radius of the tip and φ is a shape factor. Assume that the fiber tip starts sliding when an average shear stress of τ_0_ is reached when 
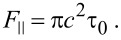
 Then, the condition below should be satisfied to prevent peeling prior to sliding:

[3]
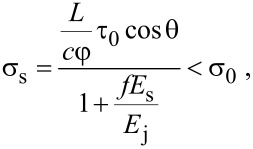


where σ_0_ represents an intrinsic tensile strength for the interface. Equation 3 can be rearranged to yield a design criterion for the ratio of the elastic modulus of the stalk *E*_s_ to the effective elastic modulus of the joint *E*_j_/*f* as

[4]
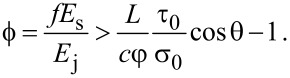


The experimental data suggest that both the soft and the very soft joint satisfy this condition. And both samples exhibit similar friction values during sliding, indicating that the elastic modulus of the joint, as long as the condition in Equation 4 is satisfied, does not affect the shear performance. The data indicates that pull-off force is affected by the flexible joint. A higher pull-off is recorded from the soft joint than the very soft joint fibers.

The terminal ends of the nanofibers at the end of the seta form a slanted plane as opposed to a horizontal one. The deformation caused in the dragging stage rotates the setae tip such that most of the fibers come in contact with the opposing surface creating adhesion and friction. In this work, the fibers tips are horizontal and form the largest possible contact with the opposing surface immediately upon contact. The default non-adhesive state and shear-initiated adhesion, similar to the gecko, is possible to obtain when the tips of the fibers are anglular as shown in [16] and [35].

## Conclusion

The results of this study show that the addition of a flexible joint, that is, a joint more flexible than the stalk of the fiber, between the tip and the stalk improves both shear and pull-off stresses. There seems to be an optimal flexibility beyond which no added benefit in shear or pull-off stress is observed. Indeed, although very soft joint fibers were able to maintain superior average shear stress at higher preload, pull-off stress was reduced compared to the soft joint fibers. We hypothesize that the improvement in performance with a flexible joint observed in this work is due to the added articulation and the resulting enhancement in the adaptability of the tip to the substrate. It is this enhancement, which reduces the tensile stress at the tip, preventing peel, and allows the tips to stay in adhesive contact during sliding. Fibers with flexible joints exhibit high pull-off stress because they maintain contact during the dragging portion of the test. One detriment of a flexible joint could be that it increases performance in both the gripping and the releasing directions, potentially diminishing the anisotropic characteristic of the adhesive. This can be remedied in the future by creating tips that are tilted. The effect of the fiber tip angle has been shown to improve anisotropy by Murphy et al. [16] and Parness et al. [23].

## Experimental

### Experimental set-up

**Figure 10:**
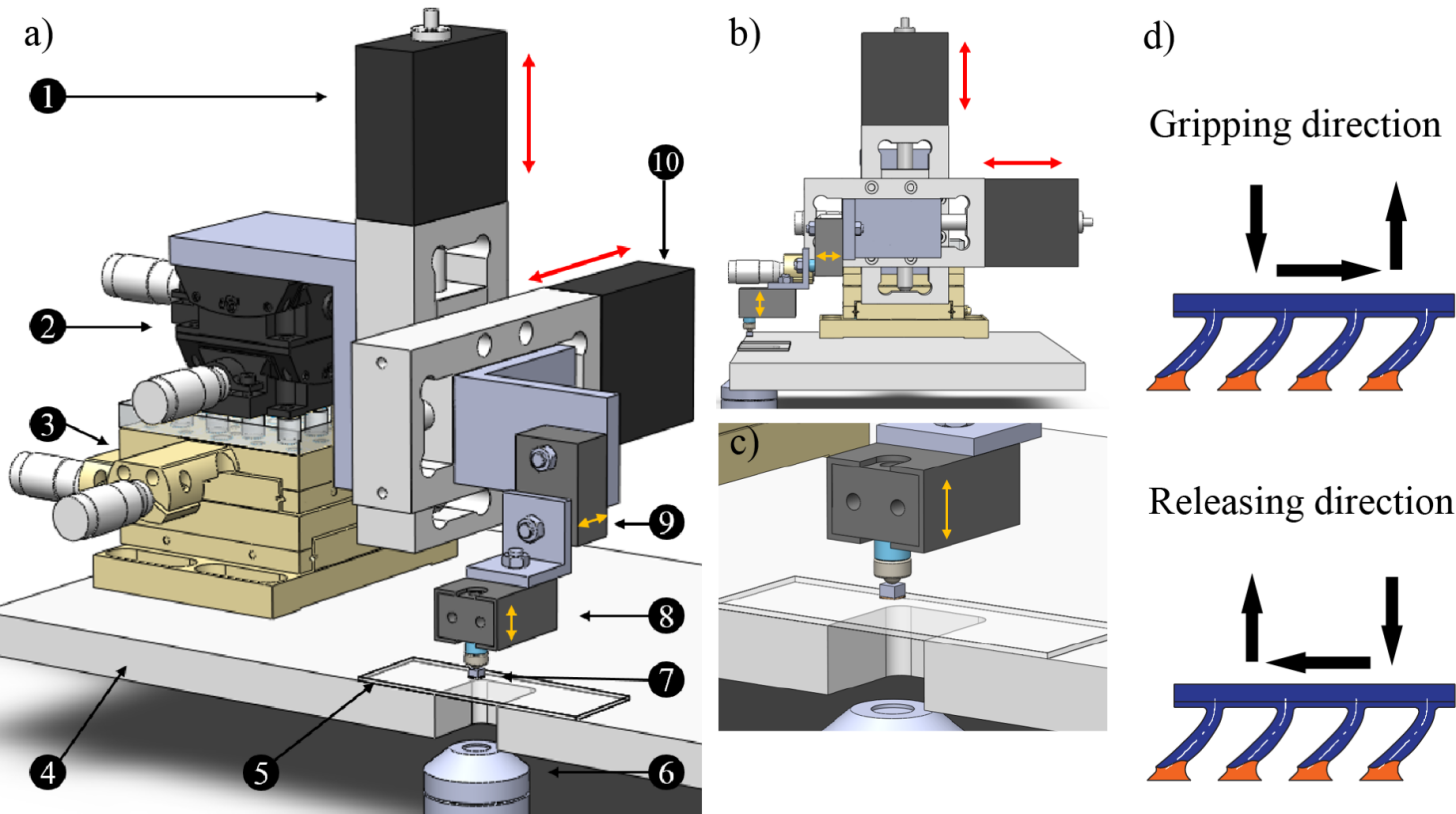
Experimental set-up used for the fabrication of bent fibers and LDP tests. a) General lay out of the set-up: 1. Motorized stage to control perpendicular to the surface movement; 2. Goniometric rotation stages to control alignment of the system; 3. Manual linear translational stages for coarse positioning; 4. Inverted microscope stage; 5. Target surface made from a glass slide; 6. Inverted microscope lens; 7. Sample; 8. Vertical load cell to read the normal force; 9. Horizontal load cell to read shear force; 10. Motorized stage to control lateral movement. b) Side view of the experimental set-up showing the movement direction of the stages. c) Detailed view of the sample attached to a steel sphere for easy alignment. d) Directions defined for the LDP tests, where gripping direction slides in favor of the actual deformation of the fibers, whereas in releasing the sliding slides against the bending of the fibers.

Both the fabrication of the bent stalk and the LDP experiments were performed using a custom setup. This setup is comprised of an inverted microscope (Eclipse MA-100, Nikon) equipped with a manual *x–y*-positioning stage (9067-XY, Newport Corp.). Two goniometers (M-GON40-L, Newport Corp.) are located on the *x–y*-positioning stage, which control roll and pitch angles. Two precision linear stages (MFA-CC, Newport Corp.) are attached to the goniometer assembly and they are configured to displace in the plane *Y–Z* where *Z* represents the perpendicular to the surface movements and *Y* represents the parallel to the surface movements. Two 500-gr load cells (GSO-500 Transducer Techniques) are attached to the precision stages, which allow forces in the *Y* and *Z* directions to be measured (Figure 10a and Figure 10b). The set-up is designed to govern stage movements in two directions – *x* and *y* – simultaneously allowing the deformation of the fibers to be visualized and aligned against the adhering surface. The output from the load cells is captured using a USB-6009 data acquisition board (National Instruments, Austin, USA) and the whole system, including the stage controller is governed by a custom Labview software that is able to control preload, contact time, displacements, velocities in the *Y–Z* plane and record the data from two load cells.

The LDP experiments were performed first by attaching the load cell stem to the acrylic peg by gluing the peg to the stem when in contact with a glass slide. After the glue is allowed to cure for 30 min, the sample is lifted away from the surface. Then the fiber sample is brought in contact with the glass substrate at 10 μm/s until a set preload is reached. This step is immediately followed by the dragging stage where the sample is displaced laterally either in the direction of the tilt (i.e., gripping direction, Figure 10d top), or opposite to tilt (i.e., releasing direction, Figure 10d bottom). After dragging the sample for 500 μm at 10 μm/s, the sample is retracted from the glass substrate. Forces in shear and normal directions are recorded as a function of time.

### Fabrication of bent fibers

**Figure 11:**
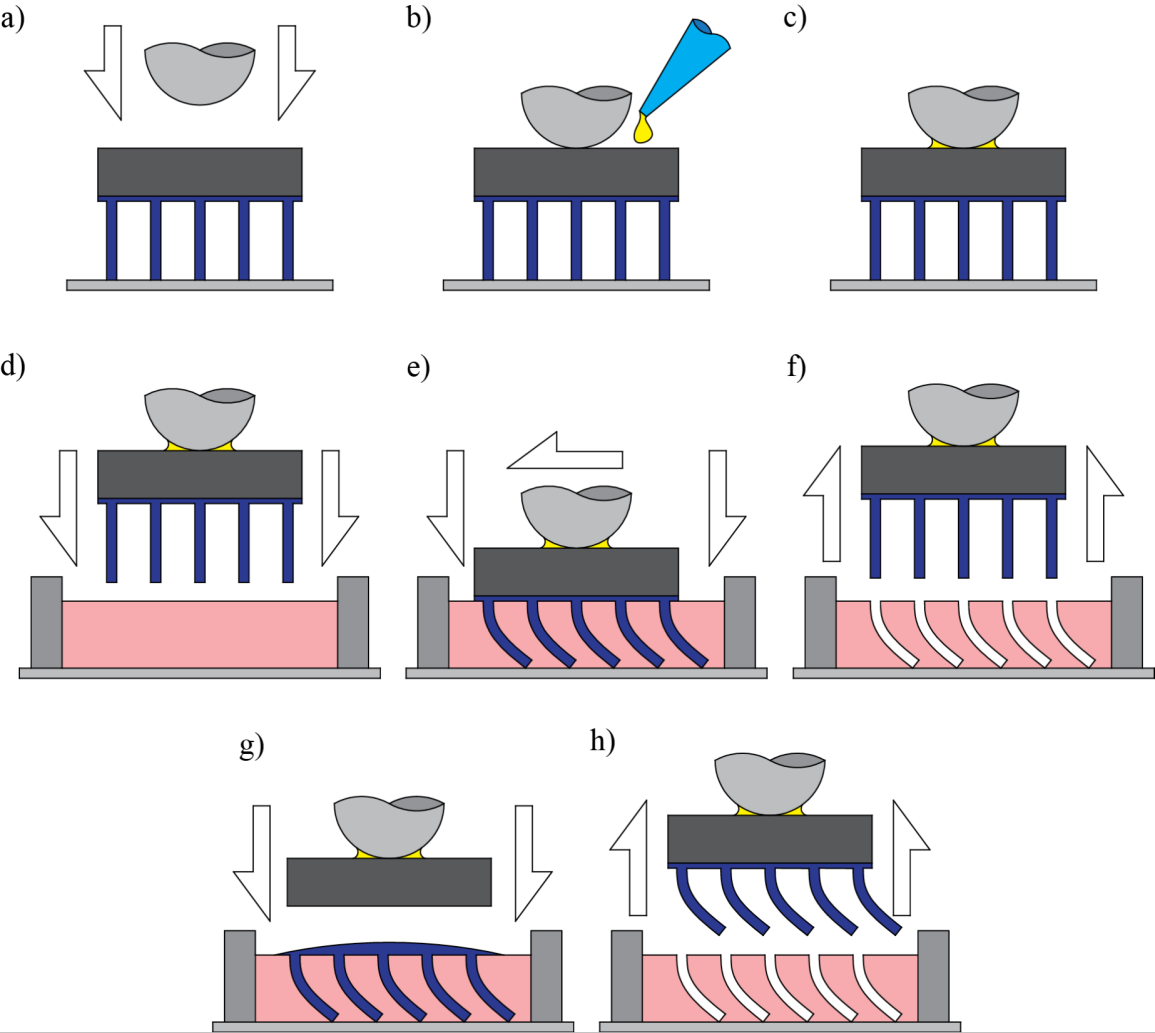
Fabrication sequence of bent fibers. a) Metal sphere brought in contact with the acrylic peg. b) The sphere is glued to the peg, c) and allowed to cure for 10 min. d) The fibers are placed into uncured silicone rubber. e) Displacement is applied in the vertical and lateral direction to create desired shape. f) The fibers are removed from the silicone rubber upon curing of the silicone rubber. g) Polyurethane is cast into the generated mold and a peg is placed on the back. h) The peg is removed from the mold, generating the final bent fibers.

Although a more detailed description about the fabrication process can be found in the work of Gorumlu et al. [36], some key steps are summarized here. The first step in the fabrication process of bent fibers is the positioning and alignment of an acrylic peg with an array of vertically aligned fibers on the experimental set-up (Figure 11a). The acrylic peg with the fiber array is placed facing down onto a glass slide previously attached to the stage on the microscope. The sphere is moved toward to the peg, allowing self-alignment of the fibers with respect to the slide. After the initial self-alignment between the microplate array and glass slide is visually checked using the inverted microscope, precision stages are used to bring the load cell stem in contact with the back of the acrylic peg. To prevent the fibers from buckling, the load cell is brought down with 1 μm steps while the load cell output is checked at each step. Once the contact is obtained, a small droplet of glue is applied to the area between stem and the back of the glass slide using needle-like tweezers (Figure 11b). The glue is allowed to dry for 10 min (Figure 11c). By using the custom Labview software, the stage is brought up in the vertical direction, whereby the microplates and the substrate are aligned but not in contact.

The second step consists of the fabrication of the master mold for the bent fibers. A small droplet of uncured silicone rubber (Moldmax 27T, Smooth On) of size similar to the peg is placed on a glass microscope slide mounted on the microscope stage, (Figure 11d). An acrylic frame can be used to retain the material. However, the high viscosity of the material and the tangential forces at the contact allow the material to be retained enough to make the mold. A small droplet of material provides a thickness of about 0.3 mm, which is more material than needed. The excess material is expelled out once the fiber array approaches the slide. Using the precision linear stage, the fibers are lowered toward the uncured silicon rubber until the fibers touch the microscope slide. Upon contact, an automated stage and the manual *x–y*-stage are used to bend the fibers to the desired shape (Figure 11e). The exact curvature of the fiber can be predicted using large beam deflection theory, which is ongoing work.

The entire process of deformation of straight fibers can be visually inspected through the inverted microscope. Once the desired shape of fibers is obtained, the system is allowed to cure for 24 h at room temperature. Note that the silicone rubber cures around the bent fibers creating a complementary mold of the bent fibers. Upon curing, the fibers are peeled from the silicon rubber mold to obtain the base master mold for bent fibers (Figure 11f). This mold is then used to cast bent fibers of a stiff polyurethane material of elastic modulus *E*_s_ = 126 MPa (Figure 11g and Figure 11h).

### Fabrication of mushroom-like fibers with joint-like elements

**Figure 12:**
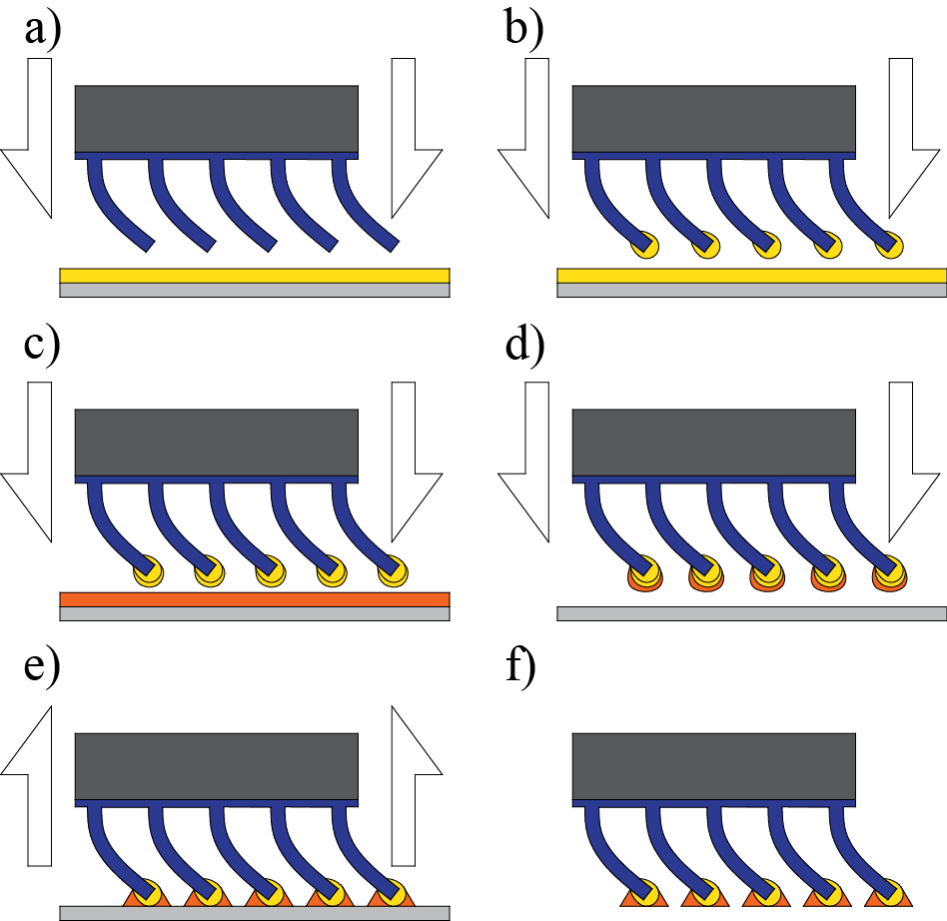
Schematic of the fabrication of soft joint-like elements and mushroom-like tips. a) The bent fiber sample is dipped into a layer of soft polyurethane. b) After curing from the first dipping step, the fibers are dipped again in a layer of the same polyurethane. c) After curing from the second dipping step, the fibers are dipped into the material that will be used to conform the tips. d) A polypropylene (PP) substrate is used to conform the mushroom-like tips. e–f) After final curing, the fibers with mushroom-like tips and joint-like elements are produced.

**Figure 13:**
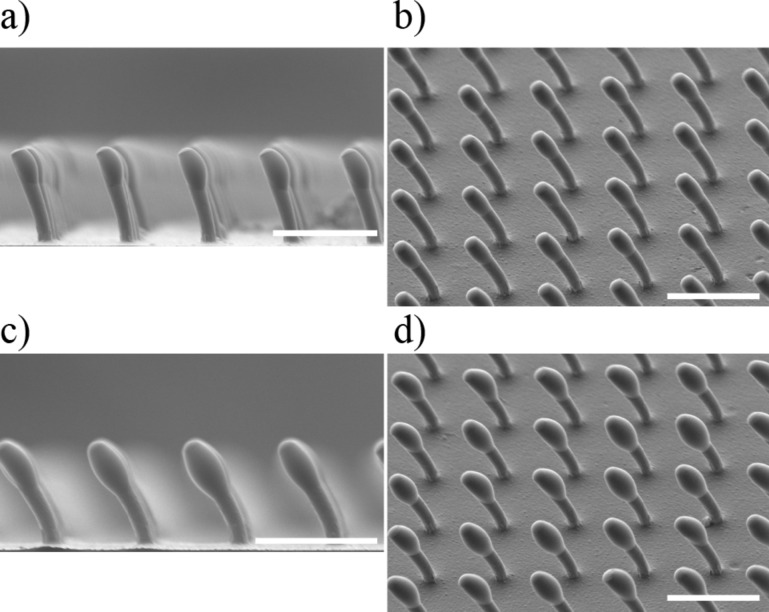
The SEM images of the fibers with flexible joints. a,b) after the first dipping step; c,d) after the second dipping step, prior to the formation of the mushroom-like tips. Scale bars: 100 µm.

The fabrication process of fibers with soft joints and mushroom-like tips is a sequence of three main steps, where a curing step is required after each step. The first step is the formation of a rounded tip at the end of the stalk as is depicted in Figure 12a and Figure 12b, where a sample with bent fibers is dipped into a glass slide with a liquid layer of soft polyurethane. A thin layer of about of 0.05 mm in thickness is generated using a spinner (WS-650 MS Spinner, Laurell Technologies, USA), in which a glass slide with the polyurethane on top is spun at 6000 rpm for 2 min. The dipping process can be repeated after a minimum curing time of 3 h (Figure 12c and Figure 12d) to increase the amount of material forming the joint. The third step is the formation of the mushroom-like tips. In this step, the same configuration of rotational speed and time used to make the rounded tips can be used for the spinner, obtaining a thin layer of about of 0.05 mm in thickness. Finally, the wet tip fiber array is placed on a polypropylene (PP) substrate to conform the mushroom-like tips, Figure 12e and Figure 12f. Although other materials can be used for the substrate, PP has shown to produce the appropriate shape for the mushroom-like tips. Figure 13 includes the SEM images of the fibers with the flexible joint material prior to forming the mushroom-like tips.

The rounding of the tip is a three step process. a) The bent fibers are dipped in a soft material to conform the basis for the joints. b) A second dipping with the same soft material enlarges the complaint tip, creating a flexible base for the tip. c) The tips are finally formed using a soft polyurethane material, following a standard method to make mushroom-like tips.

As is shown in Table 1, for all of the fiber samples, stiff stalks are made of TC-9445 (BJB Enterprises, *E* = 126 MPa) and mushroom-like tips out of M-3180 (BJB Enterprises, *E* = 8.89 MPa). The joint-like elements are made of TC-9445 for the stiff joint, M-3180 for the soft joint, and Vytaflex-30 (Smooth-On, *E* = 0.45 MPa) for the very soft joints. All the materials were cured at ambient for three days before testing.

**Table 1:** Elastic modulus (MPa) for the tip, joint and stalk for each of the studied samples.

Sample	Tip	Joint	Stalk

Stiff joint	8.89	126	126
Soft joint	8.89	8.89	126
Very soft joint	8.89	0.45	126
